# The value of the patient global health assessment in polyarticular juvenile idiopathic arthritis: a nested cohort study

**DOI:** 10.1186/s41687-021-00328-8

**Published:** 2021-06-26

**Authors:** Rebecca Trachtman, Rula Issa, Stephanie Pan, Karen M. Wilson, Daniel J. Lovell, Karen B. Onel

**Affiliations:** 1 Department of Pediatrics, Icahn School of Medicine at Mount Sinai, New York, NY USA; 2 Mindich Child Health and Development Institute, Mount Sinai, New York, NY USA; 3 Boston University, Boston, MA USA; 4 Cincinnati Children’s Hospital/University of Cincinnati, Cincinnati, OH USA; 5 Hospital for Special Surgery/Weill Cornell Medicine, New York, NY USA

## Abstract

**Objective:**

The objectives were: 1) to explore the discordance between the Patient Global Health Assessment (PtGA) scores, the Physician Global Health Assessment (PhGA) scores, and Pain scores; and 2) to explore whether the PtGA during disease remission is associated with future disease flare in pJIA.

**Methods:**

Data from an NIH funded clinical trial (NCT00792233) evaluating flare were used (*N* = 137). PtGA, PhGA, and Pain scores were assessed. Flare was defined as any active arthritis. Spearman’s correlation coefficients were calculated, and multivariable logistic regression was performed.

**Results:**

122 patients had records of flare status, of which 63 developed flare, and 42 of these patients had a visit immediately prior to flare. For study subjects with a visit immediately prior to flare, the PtGA, pain scores, and PhGA all increased at time of flare. For every unit increase in PtGA and Pain scores, there was a 9% and 23% higher odds of developing flare, respectively (*p* = 0.76, *p* = 0.40). For every unit increase in the PhGA score, there was a substantially lower odds of developing flare (*p* = 0.05).

**Conclusion:**

Our results demonstrate that the PtGA and Pain scores are strongly correlated with each other and increased at the visit prior to flare, while the PhGA scores are not. Further, the PtGA and Pain score have some predictive value for flare, while the PhGA does not. These findings highlight the value of patient input in medical care and decision-making, and support the development and use of more sophisticated PROs in the care of JIA patients.

## Introduction

Juvenile Idiopathic Arthritis (JIA) is a chronic autoimmune disease with multiple subtypes that affects approximately 300,000 children in the United States [1]. One specific subtype of disease is polyarticular JIA (pJIA), wherein more than four joints are affected with arthritis [2]. pJIA is characterized by unpredictable flares and remissions, which makes treatment decisions particularly difficult.

A validated and widely used measure of disease activity in JIA is the Juvenile Arthritis Disease Activity Score (JADAS) [3]. The JADAS is a composite disease specific outcome measure that includes: 1) the physician global assessment of current disease activity, a single question with a score of 0–10 that focuses on overall disease activity from the physician perspective; 2) the patient/parent global assessment of current overall well-being; 3) the number of joints with active arthritis; and typically 4) a modified erythrocyte sedimentation rate (ESR) or C-reactive protein (CRP); however, some versions of the JADAS do not include any laboratory measure. Data suggest that the JADAS is validated for disease activity; however, it is not a reliable measure of the global experience of children with this disease, nor does it reliably predict JIA flares [4].

In this setting, the importance of patient input and patient-reported outcomes (PROs) is increasingly recognized both in clinical care and in research, as these can provide insight beyond physician-derived measures [5, 6]. However, the only PROs routinely used in clinical practice remain the Patient Global Health Assessment (PtGA) and the Pain Visual analog scale (Pain VAS) for pain assessment. The PtGA is a single question with a score of 0–10 or 0–100 that focuses on overall health or disease activity from the patient perspective. The Pain VAS is a 10-mm or 100 mm scale that assesses pain intensity, ranging from 0 or ‘no pain’ to 10 or 100 ‘worst pain.’ Classically, the Childhood Health Assessment Questionnaire (CHAQ) has been employed, but its use has been limited by a significant floor effect, and so it has fallen out of favor [7]. Further, there is increasing interest in detailed PROs that cover specific domains, including the NIH-directed Patient Reported Outcome Measurement Information System (PROMIS), but these advanced measures require further research [8]. Although additional PROs have been evaluated for use in JIA, evidence for these tools is still lacking [9, 10].

The objectives of this study were 1) to explore the discordance between the PtGA scores, the Physician Global Health Assessment (PhGA) scores, and the Pain scores; and 2) to explore whether the PtGA during disease remission is associated with future disease flare in pJIA.

## Materials and methods

### Data source and study population

Data from an NIH funded clinical trial (NCT00792233) evaluating flare after discontinuation of anti-tumor necrosis factor (anti-TNF) therapy in pJIA were used [11]. This was a multi-center, prospective study with 2 phases conducted over a 14 month, on-protocol period. 137 patients with pJIA and clinically inactive disease were enrolled at tertiary pediatric rheumatology centers in the United States. For the first six months, patients who met the criteria for clinically inactive disease (CID) at enrollment (Wallace criteria [12]) were monitored while on stable therapy. For those who maintained CID for the entire six months, the anti-TNF agent was stopped and follow-up continued for eight more months. Other background therapy remained unchanged, and subjects were observed for the development of a protocol defined disease flare. Patients were monitored at 1, 2, 3, 4, 6 and 8 months after stopping the anti-TNF agent. The original study and this study were both approved by the institutional review boards. Patient consent for publication was obtained in the original study.

### Study variables

PtGA scores of disease impact were assessed at every study visit, on a 10-mm visual analog scale (VAS) (scored 0–10), with the question, “Considering all the ways that arthritis affects you/your child, rate how you/your child is doing IN THE PAST WEEK.” Pain was assessed at every study visit, on a 10-mm VAS (scored 0–10), with the question, “How much pain do you think you/your child has had because of your/his/her illness IN THE PAST WEEK?” PhGA scores of disease activity were assessed at every study visit, on a 10-mm VAS (scored 0–10), with the question, “Mark the line to indicate the amount of JIA disease activity TODAY.”

Disease activity was also assessed by physical examination of 71 joints. All patients began the study with clinically inactive disease as measured by the ACR Provisional Criteria [12]. Active disease, or flare, was defined for this secondary analysis, as any active arthritis, using the ACR definition of active joint, when the active joint count had been 0 at the preceding study visit [13]. Inactive disease, or ‘no flare’, was defined for this secondary analysis as an active joint count of 0. PtGA–PhGA discordance was defined as a difference between the PtGA and PhGA scores.

### Statistical analysis

Descriptive statistics were computed by ‘flare’ and ‘no flare,’ and student’s t-test, Wilcoxon rank-sum, and chi-square tests were used as appropriate. The change over time in PtGA, PhGA, and pain scores was calculated between every visit. For the first objective, Spearman’s correlation coefficients were calculated to determine the correlations and discordance between all of the global assessment scores, with the following interpretation: 0.2–0.39 ‘weak’, 0.4–0.59 ‘moderate’, 0.6–0.79 ‘strong’, and 0.8–1 ‘very strong’.

To explore whether the PtGA, PhGA and pain score was increased prior to overt flare, analysis was limited to data with a visit immediately prior to flare, defined as data recorded for the visit immediately prior to visit with flare. Finally, for patients who developed flare, a change score with the visit immediately prior to their flare and at baseline was computed. For patients who did not develop flare, we computed a change score with the last visit and at baseline. This change was used to model its association with flare status. Multivariable logistic regression was performed modeling flare as the outcome with the change in assessment score as an independent covariate, adjusting for the first visit assessment score. The area under the curve (AUC) is also reported to summarize the accuracy in delineating flare status based on change in scores, with AUC 0.5 suggesting no discrimination, 0.7 being acceptable, and 0.8–0.9 considered excellent. This was used to explore differences in the global assessment scores between study visits during remission and immediately prior to flare.

## Results

### Patients

One-hundred and twenty patients in this trial had records of their flare status. Of the 120 patients, 63 developed flare and 57 did not develop flare during the 8-month observation period after stopping the anti-TNF agent. Table 1 shows the patient characteristics of all patients, as well as those that developed flare, and those that did not. Both groups were similar with respect to age, BMI, gender, race, ethnicity, and ANA status. Surprisingly, there was a higher proportion of RF+ patients in the ‘no flare’ group, although this was not statistically significant.

**Table 1:** Baseline demographics

	All Patients (***N*** = 120)	Flare (***n*** = 63)	No Flare (***n*** = 57)	Visit Immediately Prior to Flare (***n*** = 42)	***P***-value
	Mean ± SD or Median (IQR) or N (%)	Mean ± SD or Median (IQR) or N (%)	Mean ± SD or Median (IQR) or N (%)	Mean ± SD or Median (IQR) or N (%)	
**Age, years**	11.1 ± 4.6	11.7 ± 4.9	10.4 ± 4.1	12.3 ± 4.9	0.13
**BMI**	19.3 (16.6, 21.8)	19.8 (16.4, 21.8)	18.7 (16.6, 22.2)	19.5 (16.2, 21.4)	0.79
**Sex, male**	34 (28.3)	17 (27)	17 (29.8)	14 (33.3)	0.73
**Race**					0.15
Caucasian	112 (93.3)	61 (96.8)	51 (89.5)	41 (97.6)	
Non-Caucasian	8 (6.7)	2 (3.2)	6 (10.5)	1 (2.4)	
**Ethnicity**					0.63
Hispanic or Latino	13 (10.8)	6 (9.5)	7 (12.3)	3 (7.1)	
Not Hispanic or Latino	107 (89.2)	57 (90.5)	50 (87.7)	39 (92.9)	
**ANA Status**					0.93
Positive	59 (50.4)	31 (50.8)	28 (50)	21 (52.5)	
Negative	58 (49.6)	30 (49.2)	28 (50)	19 (47.5)	
**RF Status**					0.42
Positive	14 (12)	6 (9.7)	8 (14.5)	2 (4.8)	
Negative	103 (88)	56 (90.3)	47 (85.5)	40 (95.2)	
**JIA Subtype**					0.26
Extended Oligo	18 (15)	8 (12.7)	10 (17.5)	6 (14.3)	
Poly RF+	14 (11.7)	5 (7.9)	9 (15.8)	1 (2.4)	
Poly RF-	88 (73.3)	50 (79.4)	38 (66.7)	35 (83.3)	

*SD* standard deviation, *IQR* interquartile range, *BMI* body mass index, *ANA* antinuclear antibody, *RF* rheumatoid factor, *JIA* juvenile idiopathic arthritis

### PtGA and PhGA measures

It is important to note that of the 63 study subjects that developed flare, 42 had a study visit immediately prior to flare, and so all analyses were performed for a) all study subjects with flare, and b) for study subjects with visit immediately prior to flare. Table 2 shows the PtGA, PhGA, and Pain scores at the time of the first visit that met the definition for flare and at the visit *prior* to the first visit with flare for all study subjects with flare, so that change in scores, reported as means, can be computed. This data is shown for all patients with flare, and also for those 42 patients with study visit immediately prior to flare. For all study subjects with flare, the PtGA score increased from 0.5 to 2.0, and pain increased from 0.6 to 2.3, while the PhGA increased from 0.1 at the prior visit to 2.2 at the time of first flare. For study subjects with a visit immediately prior to flare, the PtGA score increased from 0.4 to 1.6, and pain increased from 0.4 to 1.8. The PhGA, on the other hand, increased the most of all measures, from 0 at the prior visit to 2.0 at the time of first flare indication.

**Table 2:** Flare visit and prior visit

	N, all patients with flare	Mean	SD	N, patients with visit immediately prior to flare	Mean	SD
**At the time of First Flare**						
Patient Global Assessment (PtGA)	63	2.0	2.4	42	1.6	2.2
Physician Global Assessment (PhGA)	63	2.2	1.5	42	2.0	1.2
Pain	63	2.3	2.5	42	1.8	2.2
**At the visit** ***prior*** **to First Flare**^a^						
Patient Global Assessment (PtGA)	62	0.5	1.0	42	0.4	0.8
Physician Global Assessment (PhGA)	62	0.1	0.2	42	0	0.1
Pain	62	0.6	1.2	42	0.4	1.0
**Time Lapse of Visit Prior to First Flare (months)**	62	1.9	1.9			

^a^one subject had their first flare at the first visit and did not have global assessments for the prior visit

*SD* standard deviation

### Correlations of assessments for flare patients

PhGA scores at time of flare were moderately correlated with PtGA and pain scores at time of flare (*r* = 0.39 and 0.45), while the PtGA and pain scores, the two patient-reported outcomes, were highly correlated (*r* = 0.78). All correlations were highly statistically significant (*p* < 0.002).

### Predictive value of assessments

Results of multivariable logistic regression and AUC are shown in Table 3, with results for all study subjects who developed flare, as well as results for study subjects that had a visit immediately prior to development of flare. In the first analysis, for each unit increase in PtGA score, there was a 24% higher odds of developing flare (*p* = 0.37), and for each unit increase in Pain score, there was a 36% higher odds of developing flare (*p* = 0.22). For each unit increase in PhGA score, on the other hand, there was a 79% lower odds of developing flare, which was statistically significant (*p* = 0.05).

**Table 3:** Change in PtGA, pain & PhGA for all patients with flare and for patients with visit immediately prior to flare

	Odds Ratio	AUC	***p***-value
**Assessment, all patients with flare**			
Difference PtGA	1.24	0.55	0.37
Difference Pain	1.36	0.58	0.22
Difference PhGA	0.21	0.56	0.05
**Assessment, patients with visit immediately prior to flare**			
Difference PtGA	1.11	0.51	0.71
Difference Pain	1.22	0.56	0.43
Difference PhGA	0.17	0.57	0.09

*AUC* area under the curve, *PtGA* Patient Global Health Assessment, *PhGA* Physician Global Health Assessment

In the second analysis, including only study subjects with a visit immediately prior to flare, for each unit increase in PtGA score, there was an 11% higher odds of developing flare (*p* = 0.71), and for each unit increase in Pain score, there was a 22% higher odds of developing flare (*p* = 0.43). For each unit increase in PhGA score there was a substantially lower odds of developing flare (83% lower odds, *p* = 0.09). For both analyses, AUC was between 0.51–0.58 for all measures.

## Discussion

Our results show that the PtGA and Pain scores were increased at the visit prior to flare, while the PhGA scores were not. Further, the OR for flare increased with elevations in the PtGA and Pain scores, while the OR for flare was inversely related to the PhGA, indicating that patient-based measures have better but still only moderate predictive value for flare when compared to the PhGA. However, it is important to note that these analyses were underpowered and did not reach statistical significance, with the discriminant analysis revealing low AUC (close to 0.5), which suggests no discrimination. This demonstrates that simple patient-reported outcomes, in this case a global score of disease activity measured by a 10-mm VAS and a global measure of pain measured by a 10-mm VAS, capture important disease aspects in pJIA that might not be captured by the provider-based measure of overall disease activity. However, all three individual measures’ predictive values are not statistically significant, which raises the question of whether more sophisticated PROs might have stronger correlations and ability to predict flare. These findings indicate that while PROs are important for assessment of disease activity and prediction of flare, more sophisticated PROs should be developed and evaluated further, as their ability to assess disease burden and predict flare might be higher, compared to simple, non-specific patient-reported outcome measures. Further, these findings suggest that elevations in the PtGA might be an indication to monitor patients more closely, given the possibility of imminent flare.

Finally, the PtGA and Pain scores were highly correlated with each other, while they were discordant with the PhGA. This has potential implications for the JADAS score, as this composite measure utilizes both the PtGA and PhGA, and this needs to be further studied.

This study has important strengths. The data source was a well-defined patient cohort with pJIA, characterized by inactive disease at baseline, and then by relapses in a substantial number of patients. Comprehensive, repeated, standardized disease assessments were made prospectively per a clearly defined protocol, making this a rich data source for assessing the utility of outcome measures in pJIA.

The study also has some limitations. In the original NIH trial, a customized, more severe composite measure of flare was utilized. This required us to use our own specific definition of flare in order to assess the PtGA and PhGA. In addition, one explanation for the negative correlation between flare and PhGA is that in routine care physicians would have escalated treatment when they sensed impending flare; however, data for this secondary analysis were only used from the 8-month ‘anti-TNF withdrawal period,’ during which changes in treatment were not allowed as proscribed by the study protocol, and there were no known protocol violations.

These findings highlight the value of patient input in medical care and decision-making, and support the development and use of more sophisticated PROs in the care of patients with JIA.

## Data Availability

The datasets used and/or analysed during the current study are available from the corresponding author on reasonable request.

## References

[1] Thierry S, Fautrel B, Lemelle I, Guillemin F (2014). Prevalence and incidence of juvenile idiopathic arthritis: A systematic review. Joint, Bone, Spine.

[2] Petty, R. E., Laxer, R. M., Lindsley, C. B., & Wedderburn, L. B. (2015). *Textbook of Pediatric Rheumatology E-Book*. Elsevier Health Sciences.

[3] Consolaro A, Ruperto N, Bazso A, Pistorio A, Magni-Manzoni S, Filocamo G, Malattia C, Viola S, Martini A, Ravelli A, Paediatric Rheumatology International Trials Organisation (2009). Development and validation of a composite disease activity score for juvenile idiopathic arthritis. Arthritis and Rheumatism.

[4] Guzman J, Gomez-Ramirez O, Jurencak R, Shiff NJ, Berard RA, Duffy CM (2014). What matters Most for patients, parents, and clinicians in the course of juvenile idiopathic arthritis? A qualitative study. The Journal of Rheumatology.

[5] Brandon TG, Becker BG, Bevans KB, Weiss PF (2017). Patient-reported outcomes measurement information system tools for collecting patient-reported outcomes in children with juvenile arthritis. Arthritis Care & Research (Hoboken).

[6] United States Food & Drug Administration (2006). Guidance for Industry: Patient-Reported Outcome Measures: Use in Medical Product Development to Support Labeling Claims: Draft Guidance. Health and Quality of Life Outcomes.

[7] Sontichai W, Vilaiyuk S (2018). The correlation between the childhood health assessment questionnaire and disease activity in juvenile idiopathic arthritis. Musculoskeletal Care.

[8] Trachtman, R., Wang, C. M., Murray, E. T., Szymonifka, J., Pan, N., Adams, A. B., et al. (2019). PROMIS Computer Adaptive Tests and Their Correlation With Disease Activity in Juvenile Idiopathic Arthritis. *Journal of Clinical Rheumatology*, *27*(4), 131–135. 10.1097/RHU.0000000000001171. 10.1097/RHU.0000000000001171 PMC10364139 31743268

[9] Lunt LE, Shoop-Worrall S, Smith N, Cleary G, McDonagh J, Smith AD, Thomson W, McErlane F (2020). Validation of novel patient-Centred juvenile idiopathic arthritis-specific patient-reported outcome and experience measures (PROMs/PREMs). Pediatric Rheumatology Online Journal.

[10] Mosor E, Studenic P, Alunno A, Padjen I, Olsder W, Ramiro S, Bini I, Caeyers N, Gossec L, Kouloumas M, Nikiphorou E, Stones S, Wilhelmer TC, Stamm TA (2021). Young People’s perspectives on patient-reported outcome measures in inflammatory arthritis: Results of a multicentre European qualitative study from a EULAR task force. RMD Open.

[11] Lovell DJ, Johnson AL, Huang B, Gottlieb BS, Morris PW, Kimura Y, Onel K, Li SC, Grom AA, Taylor J, Brunner HI, Huggins JL, Nocton JJ, Haines KA, Edelheit BS, Shishov M, Jung LK, Williams CB, Tesher MS, Costanzo DM, Zemel LS, Dare JA, Passo MH, Ede KC, Olson JC, Cassidy EA, Griffin TA, Wagner-Weiner L, Weiss JE, Vogler LB, Rouster-Stevens KA, Beukelman T, Cron RQ, Kietz D, Schikler K, Schmidt KM, Mehta J, Wahezi DM, Ting TV, Verbsky JW, Eberhard BA, Spalding S, Chen C, Giannini EH (2018). Risk, timing and predictors of disease flare after discontinuation of anti-tumor necrosis factor therapy in children with Polyarticular forms of juvenile idiopathic arthritis with clinically inactive disease. Arthritis & Rhematology.

[12] Wallace CA, Ruperto N, Giannini EH, CARRA, PRINTO, PRCSG (2004). Preliminary criteria for clinical remission for select categories of juvenile idiopathic arthritis. The Journal of Rheumatology.

[13] Giannini, E. H., Ruperto, N., Ravelli, A., Lovell, D. J., Felson, D. T., Martini, A. (1997). Preliminary definition of improvement in juvenile arthritis. *Arthritis and Rheumatism*, *40*(7), 1202–1209. 10.1002/1529-0131(199707)40:7<1202::AID-ART3>3.0.CO;2-R 9214419

